# Lack of Association Between the *VEGFA* Gene Rs699947 Polymorphism and Periodontal Disease

**DOI:** 10.3290/j.ohpd.a43493

**Published:** 2020-02-12

**Authors:** Małgorzata Mazurek-Mochol, Ryta Łagocka, Elżbieta Dembowska, Małgorzata Kozak, Marek Sawczuk, Agnieszka Maciejewska, Damian Malinowski, Krzysztof Safranow, Andrzej Pawlik

**Affiliations:** a Assistant Professor, Department of Periodontology, Pomeranian Medical University, Szczecin, Poland. Responsible for study design, patients collection, data analysis.; b Assistant Professor, Department of Conservative Dentistry, Pomeranian Medical University, Szczecin, Poland. Responsible for patients collection and data analysis.; c Professor, Department of Periodontology, Pomeranian Medical University, Szczecin, Poland. Responsible for patients collection and data analysis.; d Assistant Professor, Department of Prosthetics, Pomeranian Medical University, Szczecin, Poland. Responsible for patients collection and data analysis.; e Professor, Department Faculty of Physical Education and Health Promotion, University of Szczecin, Szczecin, Poland. Responsible for genetic analysis.; f Professor, Department Faculty of Physical Education and Health Promotion, University of Szczecin, Szczecin, Poland. Responsible for genetic analysis.; g Research Assistant, Department of Pharmacology, Pomeranian Medical University, Szczecin, Poland. Responsible for genetic analysis.; h Assistant Professor, Department of Biochemistry and Medical Chemistry, Pomeranian Medical University, Szczecin, Poland. Responsible for statistical analysis.; i Professor, Department of Physiology, Pomeranian Medical University, Szczecin, Poland. Responsible for study design and manuscript preparation.

**Keywords:** gene, polymorphism, periodontal disease, VEGFA

## Abstract

**Purpose:**

Periodontal disease is a chronic inflammatory disease characterised by the infiltration of inflammatory cells as well as activation of pathological angiogenesis in gingival tissues. Vascular endothelial growth factor (VEGF) plays a statistically significant role in the regulation of angiogenesis and induction of an inflammatory response in periodontal tissues.

**Materials and Methods:**

We examined the association between the *VEGFA* gene rs699947 polymorphism and periodontal disease. This study enrolled 200 patients with periodontal disease (130 non-smokers and 70 smokers) and 160 control subjects (126 non-smokers and 34 smokers).

**Results:**

There were no statistically significant differences in the distribution of* VEGFA* rs699947 genotypes and alleles between patients with periodontal disease and control subjects, also in the case when the analysis was performed in subgroups stratified according to smoking status.

**Conclusion:**

The results of this study suggest there is no association between the *VEGFA* gene rs699947 polymorphism and periodontal disease.

Periodontal disease is a chronic inflammatory disease characterised by the infiltration of inflammatory cells as well as activation of pathological angiogenesis in gingival tissues. This disease is associated with bacterial infections. The immune response to bacteria can lead to initiation of the disease and consequently to the destruction of gingival tissues and bone resorption.^2,8,19^ Numerous growth factors and cytokines are involved in this process, including tumour necrosis factor α, interleukin (IL)-1, IL-6, IL-8, platelet-derived growth factor, fibroblast growth factor, prostaglandin E and endotoxins. These mediators stimulate the synthesis of vascular endothelial growth factor (VEGF).^14^ VEGF plays an important role in the regulation of angiogenesis in healthy gingival tissue as well as in pathologic conditions such as periodontal disease, increasing the expansion of the vascular network.^2,14,16^

VEGF is secreted by mesenchymal cells, whereas VEGF receptors are present on vascular endothelial cells, monocytes, macrophages and pericytes.^11,17^ In periodontal disease, VEGF receptors are expressed on fibroblasts and increased levels of VEGF have been detected in gingival crevicular fluid of patients. Moreover, VEGF regulates endothelial cell migration and vascular permeability. VEGF is also involved in bone resorption and stimulates nitric oxide production.^9^ It has been shown that VEGF plays a statistically significant role in the inflammatory response in gingival tissue in periodontal disease. Blocking VEGF expression decreases the inflammatory response in inflamed connective tissue in periodontal disease. The inhibition of cyclooxygenase-2 in a rat model of periodontitis resulted in decreased expression of VEGF mRNA.^13^

Several polymorphisms have been detected in VEGF and have been examined in various inflammatory diseases. In this study, we examined the association between the *VEGF* gene rs699947 polymorphism and periodontal disease.

## Material and Methods

### Study Subjects

This cross-sectional study enrolled 360 Caucasian subjects (with an age range of 25–69 years) from the West Pomeranian region of Poland. The subjects were submitted to anamnesis and to clinical and periodontal examination. Then the subjects were divided into two subgroups: patients with periodontal disease, and healthy subjects without periodontal disease. The first group was comprised of 200 patients (87 men, 113 women), aged 26–69 years (mean 50.47 ± 9.09), with chronic periodontal disease, diagnosed using the periodontal disease classification system of the American Academy of Periodontology.^1^ Patients diagnosed with chronic generalised moderate-advanced periodontitis had a periodontal involvement of at least 30% and a clinical attachment loss of ≥ 3 mm. Of this group of 200 patients, 130 were non-smokers and 70 were smokers. Those with no evidence of clinical features of periodontal disease (a periodontally healthy individual was defined by clinical attachment loss <1 mm) were categorised as healthy and were considered the control group (160 subjects; 61 men and 99 women; aged 25–69 years; mean 42.97 ± 11.22). In the healthy group, 126 subjects were non-smokers and 34 were smokers.

Patients were enrolled by a clinical researcher based on the following inclusion criteria: four or more periodontal pockets with a probing depth ≥ 5 mm and bleeding on probing. Clinical parameters were recorded at six sites per tooth.

Exclusion criteria included systemic disease, patients who used systemic or subgingival antimicrobial agents, or were chronic users of anti-inflammatory medication. Subjects were also excluded from the study if they had a history of hepatitis, AIDS or HIV, recent radiation therapy, diabetes, uncontrolled hypertension, use of immunosuppressive medications or were pregnant.

Additionally, the subjects were stratified into smoking and non-smoking subgroups to analyse potential interaction between smoking and genotype in their influence on periodontal disease risk. Patients with or without periodontitis who had smoked tobacco for at least 5 years without interruption and smoked 10 cigarettes or more per day were allocated to the smoking subgroup. Patients with or without periodontitis who had never smoked were placed in the non-smoking subgroup. All patients were otherwise healthy and were not subjected to periodontal treatment or antibiotics for at least 6 months before the study.

The study was approved by the ethics committee at Pomeranian Medical University, Szczecin, Poland, (BN-001/93/ 08) and written informed consent was obtained from all subjects.

### Periodontal Examination

Periodontal evaluation included probing pocket depth (PD), clinical attachment loss (CAL), the approximal plaque index (API), and modified sulcus bleeding index (% SBI).

Clinical measurements were taken in standard conditions in a dental clinic. PD and CAL were assessed at six sites per tooth (disto-, mesiobuccal, midbuccal, disto-, mesiolingual/palatal, and midlingual/midpalatal), using a periodontal probe calibrated to 1 mm. PD represents the distance from the gingival margin to the bottom of the periodontal pocket and CAL represents the distance from the cementoenamel junction to the bottom of the periodontal pocket. A UNC-15 colour-coded probe (Hu-Friedy Mfg Co, Chicago, IL, USA) (graduated 1-2-3-4-5-6-7-8-9-10-11-12-13-14-15) was used for all explorations. Pressure of approximately 20 g was applied for probing.

### Genotyping

All samples were genotyped in duplicate using allelic discrimination assays with TaqMan probes (Applied Biosystems, Carlsbad, CA, USA) on a 7500 Fast Real-Time PCR Detection System (Applied Biosystems).

### Statistical Analysis

The consistency of the genotype distribution with Hardy–Weinberg equilibrium (HWE) was assessed using with Fisher’s exact test. Chi-squared and Fisher’s exact tests were used to compare genotype and allele distributions between groups. Multivariate logistic regression with rs699947 genotype, age, sex, body mass index (BMI), smoking, education level and salary level as independent variables was used to find independent risk factors of periodontitis. The power of the study to detect an association of the rs699947 SNP with periodontitis was estimated using the PS program version 3.0.43. The study sample size was sufficient to detect with 80% probability the true effect size of the association, measured as odds ratio (OR) equal to 0.526 or 2.101 for dominant inheritance model (CC+AC vs AA), 0.352 or 2.134 for recessive model (CC vs AA+AC), and 0.640 or 1.541 for additive model (C vs A). A p value of <0.05 was considered to indicate a statistically significant result.

## Results

The clinical periodontal parameters in the studied groups are shown in Table 1. The distribution of *VEGFA* rs699947 genotypes among patients with periodontal disease and control subjects was present in HWE and is shown in Table 2. There were no statistically significant differences in the distribution of *VEGFA* rs699947 genotypes and alleles between patients with periodontal disease and control subjects.

**Table 1:** The clinical periodontal parameters of studied subjects

Parameter	Controls (n = 160)	Periodontitis patients (n = 200)
SEX (M/F)	55/105	84/116
AGE years (mean ± SD)	45.28 ± 10.15	49.85 ± 8.71
API % (mean ± SD)	35.81 ± 20.66	72.98 ± 21.03
SBI % (mean ± SD)	6.53 ± 11.29	57.66 ± 25.45
PPD mm (mean ± SD)	1.63 ± 0.54	4.36 ± 2.32
CAL mm (mean ± SD)	0.41 ± 1.18	5.04 ± 2.41

API, approximal plaque index; SBI, sulcus bleeding index; PPD, probing pocket depth; CAL, clinical attachment loss.

**Table 2:** The distribution of *VEGFA* rs699947 genotypes in periodontal disease patients and control group

	PD patients		Control group		p^a^		p^b^	OR (95% CI)
	n	%	n	%				
*VEGFA rs699947*								
*genotype*								
AA	55	27.50%	48	30.00%	0.51	CC+AC vs AA	0.64	1.13 (0.71–1.79)
AC	103	51.50%	86	53.75%		CC vs AC+AA	0.28	1.37 (0.80–2.35)
CC	42	21.00%	26	16.25%		CC vs AA	0.34	1.41 (0.76–2.63)
						AC vs AA	0.90	1.05 (0.65–1.69)
						CC vs AC	0.32	1.35 (0.77–2.38)
*VEGFA rs699947*								
*Allele*								
A	213	53.25%	182	56.88%				
C	187	46.75%	138	43.12%		C vs A	0.37	1.16 (0.86–1.56)

^a^χ^2^ test; ^b^ Fisher exact test; *VEGFA* rs699947, HWE, PD patients p = 0.67; control group p = 0.26.

Additionally, we performed analysis stratified according to smoking status and compared the distribution of studied polymorphisms between smoking patients with periodontal disease and smoking control subjects, and between non-smoking patients with periodontal disease and non-smoking control subjects. As shown in Tables 3 and 4, these differences were not statistically significant.

**Table 3:** The distribution of *VEGFA* rs699947 genotypes in periodontal disease patients and control group in non-smokers’ group

	PD patients		Control group		p^a^		p^b^	OR (95% CI)
	(non-smokers)		(non-smokers)					
	n	%	n	%				
*VEGFA rs699947*								
*genotype*								
AA	35	26.92%	39	30.95%	0.62	CC+AC vs AA	0.49	1.22 (0.71–2.09)
AC	69	53.08%	67	53.18%		CC vs AC+AA	0.42	1.33 (0.70–2.52)
CC	26	20.00%	20	15.87%		CC vs AA	0.35	1.45 (0.69–3.04)
						AC vs AA	0.67	1.15 (0.65–2.02)
						CC vs AC	0.61	1.26 (0.6–2.47)
*VEGFA rs699947*								
*Allele*								
A	139	53.46%	145	57.54%				
C	121	46.54%	107	42.46%		C vs A	0.37	1.18 (0.83–1.67)

^a^ χ^2^ test; ^b^ Fisher exact test.

**Table 4:** The distribution of *VEGFA* rs699947 genotypes in periodontal disease patients and control group in smokers’ group

	PD patients		Control group		p^a^		p^b^	OR (95% CI)
	(smokers)		(smokers)					
	n	%	n	%				
*VEGFA rs699947*								
*genotype*								
AA	20	28.57%	9	26.47%	0.75	CC+AC vs AA	1.00	0.90 (0.36–2.26)
AC	34	48.57%	19	55.88%		CC vs AC+AA	0.62	1.38 (0.49–3.93)
CC	16	22.86%	6	17.65%		CC vs AA	1.00	1.20 (0.35–4.08)
						AC vs AA	0.81	0.81 (0.31–2.12)
						CC vs AC	0.59	1.49 (0.50–4.45)
*VEGFA rs699947*								
*Allele*								
A	74	52.86%	37	54.41%				
C	66	47.14%	31	45.59%		C vs A	0.88	1.07 (0.60–1.90)

^a^ χ^2^ test; ^b^ Fisher exact test.

A multivariate logistic regression model (Table 5) showed that older age, higher BMI and smoking were statistically significant independent risk factors of periodontitis, while sex, socioeconomic status (education, salary) and rs699947 genotype were not statistically significantly associated with periodontitis.

**Table 5:** Multivariate logistic regression analysis for the risk of periodontitis with age, sex, BMI, smoking status, education level, salary level and *VEGFA* rs699947 genotype as independent variables

Independent variables	OR (95% CI)	p
Age (years)	1.043 (1.018–1.070)	0.00091
Sex (male vs female)	1.09 (0.67–1.77)	0.71
BMI (kg/m^2^)	1.21 (1.10–1.34)	0.00017
Smoking (yes vs no)	2.20 (1.31–3.68)	0.0028
Education level ^a^	1.11 (0.64–1.90)	0.71
Salary level ^b^	1.01 (0.64–1.59)	0.96
*VEGFA* rs699947 (number of C alleles)	1.25 (0.90–1.74)	0.19

^a^Educational level: 1, primary + junior (<11 years of learning); 2, secondary (15 years of learning); 3 higher (>15 years of learning); ^b^Salary level: 1, low; 2, average; 3, high.

## Discussion

In this study, we examined the association between the VEGF gene rs699947 polymorphism and periodontal disease. Our results suggest a lack of association between this polymorphism and periodontal disease both in smoking and non-smoking patients. Periodontal disease is the chronic inflammatory status induced by various proinflammatory cytokines, chemokines and other mediators. VEGF is an important angiogenic mediator that plays a statistically significant role in the induction and maintenance of the inflammatory response in periodontal tissue. The role of VEGF in the pathogenesis of periodontal disease has been investigated in animal models and in clinical studies.^3,6,7^ Numerous studies have indicated increased expression of VEGF in patients with periodontal disease.^2,5,18^

Vladau et al indicated that VEGF plays a key role in the initiation of periodontal disease, enhancing angiogenesis and the inflammatory response in periodontal tissue. VEGF expression in the gingival epithelium was positively correlated with the severity of periodontal disease.^20^ Sreedhara et al examined the effect of periodontal therapy on VEGF concentration in gingival crevicular fluid. These authors have indicated that the concentration of VEGF in gingival crevicular fluid was significantly correlated with both periodontal disease progression and healing after therapy.^14^ Miyagawa et al examined the localisation of VEGF in rat periodontal tissues during experimental tooth movement in vivo, and the effects of continuous compressive force on VEGF production and angiogenic activity in human periodontal ligament cells in vitro. These results indicate that continuous compressive force enhances VEGF production and angiogenic activity in periodontal ligament cells, which may contribute to periodontal remodelling, including angiogenesis, during orthodontic tooth movement.^10^

Several studies have investigated the factors regulating VEGF expression in periodontal disease. Ramya et al and Guneri et al have shown that diabetes may enhance the expression of VEGF in patients with periodontal disease. The expression of VEGF was significantly higher in patients with diabetes and periodontal disease than in non-diabetic subjects with chronic periodontitis.^4,15^ Vasconcelos et al showed that in periodontal disease VEGF expression may be regulated by various factors, such as hypoxia, bacterial endotoxins and inflammatory cytokines.^19^ In addition, a previous study suggests that hypoxia may enhance VEGF expression in periodontal tissue.^12^ The results of our study suggest a lack of association between the *VEGFA* gene rs699947 polymorphism and periodontal disease. It is likely that other factors, such as proinflammatory cytokines and other mediators, various metabolites, hypoxia and bacterial endotoxins play a more important role in the regulation of VEGF expression in periodontal disease.

## Conclusion

The results of this study suggest a lack of association between *VEGFA* gene rs699947 polymorphism and periodontal disease.
